# Artificial intelligence-enabled deep clinical phenotyping in heart failure with preserved ejection fraction: from patient subtypes to precision care

**DOI:** 10.3389/fcvm.2026.1956165

**Published:** 2026-10-06

**Authors:** Francisco Epelde

**Affiliations:** 1 Department of Internal Medicine, Parc Taulí Hospital Universitari, Sabadell, Spain; 2 Institut d’Investigació i Innovació Parc Taulí (I3PT-CERCA), Sabadell, Spain; 3 Department of Medicine, Universitat Autònoma de Barcelona, Barcelona, Spain

**Keywords:** artificial intelligence, clinical trials, deep phenotyping, experimental models, heart failure with preserved ejection fraction, machine learning, multi-omics, precision medicine

## Abstract

Heart failure with preserved ejection fraction (HFpEF) is a clinical syndrome defined by shared manifestations but produced by diverse combinations of cardiometabolic, vascular, inflammatory, renal, pulmonary, atrial, skeletal-muscle, and aging-related mechanisms. This heterogeneity limits the value of classifications based only on left ventricular ejection fraction and helps explain why many mechanistically plausible interventions have produced neutral average results. Deep phenotyping can move the field from descriptive heterogeneity toward biologically coherent and clinically actionable subtypes by integrating clinical trajectories, advanced imaging, exercise and invasive hemodynamics, circulating biomarkers, multiomics, and digital physiology. Artificial intelligence (AI) offers methods to combine these high-dimensional data, but statistical clusters should not be equated with disease endotypes. A useful subtype must be reproducible across cohorts, anchored to plausible biology, associated with a distinct clinical course, and able to improve diagnosis, therapeutic selection, or clinical-trial design. This Review examines the translational continuum from experimental HFpEF models to human patient subtypes, summarizes the main deep-phenotyping domains and AI approaches, and proposes seven clinically interpretable subtype families: cardiometabolic-inflammatory, hypertensive-vascular aging, atrial myopathy, pulmonary vascular-right ventricular, cardiorenal-congestive, peripheral-frailty, and specific etiologic phenocopies. Current evidence supports a layered treatment strategy in which broadly effective therapies, particularly sodium-glucose cotransporter 2 inhibitors, are combined with phenotype-directed management of obesity, diabetes, mineralocorticoid receptor activation, atrial fibrillation, congestion, pulmonary vascular disease, and physical deconditioning. The central next step is prospective validation of dynamic subtype assignments in external populations and phenotype-enriched trials. Phenogroup labels should therefore be regarded as pragmatic, transitional proxies for dominant mechanisms when those mechanisms cannot yet be measured directly at the individual-patient level; their value lies in improving decisions while the field moves toward fully individualized mechanism-based treatment. Deep phenotyping will be clinically valuable only when it changes a decision and improves an outcome, rather than merely increasing descriptive complexity.

## 1 Introduction

Heart failure with preserved ejection fraction (HFpEF) is not a single myocardial disease. It is a syndrome in which symptoms and signs of heart failure, elevated filling pressures, and preserved left ventricular ejection fraction arise from variable combinations of myocardial, vascular, renal, pulmonary, metabolic, inflammatory, and peripheral abnormalities (1, 2). Contemporary definitions and guidelines have improved diagnostic consistency and established effective therapies, but the diagnostic label still groups together patients with markedly different causal pathways and trajectories (3–5). The clinical consequences of this heterogeneity are potentially important: although broadly effective therapies such as sodium-glucose cotransporter 2 inhibitors show consistent benefit across the HFpEF spectrum, the magnitude, mechanism, and patient-centered relevance of benefit from other interventions may differ across dominant phenotypes. Phenotyping should therefore complement, rather than delay or replace, therapies with established population-wide efficacy. The limitations of an ejection fraction-centered taxonomy are most visible in patients with exertional dyspnea, obesity, atrial fibrillation, chronic kidney disease, pulmonary hypertension, or apparently normal natriuretic peptide concentrations. Diagnostic frameworks such as HFA-PEFF and H2FPEF provide useful probabilistic structure, yet they were designed primarily to establish the likelihood of HFpEF rather than to define its causal endotypes (6–8). The next clinical question is therefore not only whether HFpEF is present, but which mechanisms dominate in a particular patient, how those mechanisms interact, and whether that information changes management. Phenotype-based care has long been proposed as a pragmatic response to HFpEF heterogeneity (9, 10). However, the field has frequently used phenotype, phenogroup, endotype, and cluster as interchangeable terms. A phenotype is an observable pattern; a phenogroup is a data-derived group of patients who share measured characteristics; an endotype is a subgroup defined by a distinct functional or pathobiological mechanism. These distinctions matter. A cluster that predicts hospitalization may be clinically useful for risk stratification, but it does not become an endotype unless it has mechanistic coherence and, ideally, predicts differential benefit from a targeted intervention. This Review focuses on the translational pathway requested by the Research Topic: aligning mechanisms derived from experimental models with deeply characterized human cohorts, using AI to identify reproducible patient subtypes, and converting those subtypes into precision care and enriched clinical trials. The central argument is that deep phenotyping should be judged by decision utility. More measurements and more complex algorithms are not inherently superior. The clinically relevant endpoint is a subtype assignment that is reliable, interpretable, dynamic when biology changes, and capable of improving a diagnostic or therapeutic decision.

## 2 Scope and literature approach

This narrative Review was developed through a targeted search of PubMed/MEDLINE and major guideline and publisher sources up to 29 July 2026. Search concepts included HFpEF combined with phenotyping, phenomapping, machine learning, artificial intelligence, imaging, exercise hemodynamics, biomarkers, proteomics, metabolomics, transcriptomics, single-cell analysis, experimental models, obesity, atrial myopathy, pulmonary vascular disease, cardiorenal disease, and precision therapy. Priority was given to peer-reviewed studies published from 2020 onward, supplemented by landmark earlier studies required to explain the development of diagnostic scores, phenomapping, experimental models, and pivotal treatment trials. Because the objective was conceptual and translational rather than an exhaustive estimate of effect, study selection was not performed as a systematic review and no formal meta-analysis was undertaken. The Review uses a deliberately stringent definition of an actionable HFpEF subtype. Four requirements are proposed: reproducibility in an external population; biological anchoring through physiology, molecular data, or cross-species evidence; temporal meaning, including stability or predictable evolution; and clinical utility demonstrated by a change in diagnosis, treatment, trial eligibility, or outcome. These requirements are used throughout the article to distinguish mature evidence from hypothesis-generating classification.

## 3 What constitutes deep phenotyping in HFpEF?

Deep phenotyping is the coordinated acquisition and integration of measurements that describe disease expression at multiple biological scales. In HFpEF, it should extend from the patient's longitudinal clinical course to organ-level physiology and cellular or molecular mechanisms. Large prospective resources, including the United Kingdom HFpEF Registry, illustrate the value of standardized multimodal cohorts designed to support endotype discovery rather than retrospective extraction of whatever variables happen to be available (11). Table 1 summarizes the principal domains.

**Table 1:** Core domains of deep phenotyping in HFpEF.

Domain	Representative measures	Mechanistic signal	Potential clinical use	Principal limitations
Clinical/longitudinal	Comorbidities, symptoms, BP, rhythm, medications, congestion/event trajectories	Pre-test probability; evolving context	Select next tests; identify drivers; distinguish stable vs. decompensated	Documentation variability; treatment confounding; static snapshots
Imaging	Echo geometry, diastolic indices, strain, LA/RV function; CMR tissue characterization	Remodeling, chamber interaction, fibrosis/infiltration	Etiologic diagnosis; organ-level subtype; prognosis	Load dependence; vendor/site variability; opaque latent features
Exercise/hemodynamics	CPET, exercise echo, pressure-flow, cardiac output reserve, oxygen extraction	Central vs. peripheral limitation; latent filling-pressure/pulmonary vascular abnormality	Resolve uncertain diagnosis; enrich trials	Resource intensive; referral bias; protocol heterogeneity
Biomarkers/multi-omics	Natriuretic peptides; injury/fibrosis/inflammation; proteomics, metabolomics, genomics	Pathway activity; molecular endotypes	Risk stratification; pathway matching; cross-species alignment	Batch effects; reverse causation; cost; limited external validation
Tissue/single-cell	Myocardial/adipose/vascular tissue; single-cell/spatial transcriptomics	Cell states; cell-cell communication; spatial biology	Mechanistic anchoring; target discovery	Selected samples; cross-sectional design; limited scalability
Digital physiology	Wearables; rhythm burden; activity; sleep; weight; BP; patient-reported outcomes	Dynamic reserve; early decompensation	Longitudinal reassessment; remote monitoring	Adherence/access bias; non-cardiac influences; governance

CMR, cardiovascular magnetic resonance; CPET, cardiopulmonary exercise testing; HFpEF, heart failure with preserved ejection fraction; LA, left atrial; RV, right ventricular.

Failure with preserved ejection fraction; LA, left atrial; RV, right ventricular. Clinical phenotyping remains the indispensable first layer. Age, sex, blood pressure history, obesity distribution, diabetes, chronic kidney disease, atrial fibrillation, coronary disease, pulmonary disease, sleep-disordered breathing, anemia, frailty, and medication exposure provide mechanistic priors. Yet static comorbidity counts are insufficient. Recurrent congestion, rapid diuretic escalation, transition from exercise-only elevation of filling pressure to resting congestion, incident atrial fibrillation, renal decline, and progressive loss of functional capacity can reveal trajectories that cross conventional phenotype boundaries. A dynamic phenotype should therefore include time-stamped events and treatment responses rather than a single baseline snapshot. Cardiac imaging provides structural and functional detail beyond ejection fraction. Standard echocardiography should include chamber geometry, left atrial volume and function, diastolic indices, right ventricular function, pulmonary pressure estimates, valvular disease, and pericardial constraint. Speckle-tracking measures can expose subclinical longitudinal systolic dysfunction and left atrial or right ventricular mechanical impairment. Cardiovascular magnetic resonance adds myocardial tissue characterization, extracellular volume, focal scar, infiltration, pericardial anatomy, and more reproducible assessment of ventricular mass and volumes. Nevertheless, an abnormal imaging variable is not automatically causal; it must be interpreted within loading conditions and the wider phenotype. Resting measurements often underestimate HFpEF physiology. Exercise echocardiography, cardiopulmonary exercise testing, and invasive exercise hemodynamics can identify patients whose filling pressures are normal at rest but rise abnormally during exertion (12, 13). Simultaneous assessment of cardiac output reserve, pulmonary artery pressure-flow relationships, right-sided reserve, chronotropic response, ventilation, oxygen extraction, and skeletal-muscle performance can distinguish central from peripheral limitation. Impaired myocardial mechanics despite preserved ejection fraction also carries prognostic information, emphasizing that preserved ejection fraction does not mean preserved systolic performance (14). The main barrier is scalability: high-quality exercise phenotyping is resource intensive and remains concentrated in specialist centers. Recent exercise-imaging data further illustrate why stress testing can reveal physiology that is invisible at rest. In a 2025 study, Fazzini et al. characterized patients with a paradoxical exercise-induced reduction in left ventricular ejection fraction despite the absence of coronary artery disease. The cohort was predominantly female and showed subsequent risk of atrial fibrillation and heart-failure hospitalization, suggesting that abnormal systolic reserve during exertion may identify a clinically relevant preclinical phenotype not apparent on resting assessment. These findings support the use of exercise imaging to distinguish impaired systolic reserve, diastolic reserve, or combined abnormalities in patients with otherwise unexplained exertional symptoms (15). Molecular phenotyping can connect clinical expression to pathway activity. Inflammatory and endothelial signals, extracellular matrix turnover, myocardial injury, neurohormonal activation, renal dysfunction, and metabolic stress are all relevant, but single biomarkers rarely have adequate specificity (16). Epicardial adipose tissue is a useful example of an intermediate phenotype because imaging- defined burden can be linked to local inflammatory and metabolic biology, coronary microvascular dysfunction, and obesity-related hemodynamics (17). Proteomic and metabolomic panels may reveal latent pathways that are not apparent from conventional variables, while genomics can identify causal predisposition rather than secondary disease consequences. Single-cell and spatial technologies are beginning to resolve cellular heterogeneity within the human HFpEF myocardium. A 2026 human single-cell analysis demonstrated a distinct cell-type-specific transcriptomic landscape and highlighted incomplete correspondence between human disease and available experimental models (18). Complementary single-cell work has identified differences between HFpEF and reduced-ejection-fraction heart failure in cardiomyocyte and non-cardiomyocyte states (19). These studies are important mechanistic anchors, but myocardial tissue is obtained from highly selected populations and cross-sectional tissue cannot by itself establish the sequence of disease development. Digital phenotyping adds repeated measurements outside the clinic. Activity, heart rate, rhythm burden, sleep, weight, blood pressure, respiratory rate, and patient-reported symptoms can identify decompensation or loss of reserve before a conventional visit. The strength of digital data is density over time; the weakness is context. Changes may reflect behavior, device adherence, social conditions, or noncardiac illness. Digital features should therefore complement, not replace, clinical interpretation and should be analyzed with explicit attention to missingness and unequal access.

## 4 Experimental models as mechanistic anchors

Experimental models are essential for causal inference, perturbation testing, and tissue-level analysis, but no single model reproduces the entire HFpEF syndrome. The most productive strategy is mechanism-to-phenotype alignment: a model should be selected because it reproduces a defined pathway or physiological feature that can be measured in humans, not because it has a preserved ejection fraction and one marker of diastolic dysfunction. Reviews comparing humans and mice have emphasized the need to embrace clinical complexity while avoiding a checklist approach that rewards superficial similarity (20). The comorbidity-driven paradigm links obesity, diabetes, hypertension, and aging to systemic inflammation, coronary microvascular endothelial dysfunction, impaired nitric oxide-protein kinase G signaling, cardiomyocyte stiffness, and interstitial fibrosis (21). Multi-hit rodent models have operationalized this concept. High-fat diet combined with nitric oxide synthase inhibition produces a phenotype with obesity, hypertension, metabolic dysfunction, exercise intolerance, and preserved ejection fraction; mechanistic work identified inducible nitric oxide synthase-dependent nitrosative stress and suppression of the unfolded protein response as potential drivers (22). Multi-hit models are more clinically recognizable than isolated pressure overload, but the intensity and timing of insults can create biology that differs from slowly evolving human disease. Preclinical intervention studies illustrate both the opportunity and the risk of reverse translation. In a multihit mouse model, liraglutide and dapagliflozin produced distinct effects on cardiac structure and function (23). Such studies can test whether a clinically observed responder phenotype has a plausible mechanistic basis. However, concordance should be assessed across multiple readouts—symptoms or exercise capacity, hemodynamics, tissue biology, and biomarkers—rather than by normalization of a single echocardiographic parameter. Single-cell profiling can define model-specific cellular states. Fibroblast activation patterns in experimental HFpEF differ from those in pressure-overload or systolic heart failure models, suggesting that fibrosis should not be treated as one uniform pathway (24). Broader work on immune-fibroblast communication in human and experimental heart failure further supports the concept that cell-cell signaling networks, rather than isolated cell abundance, may provide more transferable therapeutic targets (25). The growing availability of human single-cell data creates an opportunity to score experimental models according to their molecular similarity to specific human subtypes. Large-animal models add clinically relevant chamber size, coronary anatomy, pressure-volume relationships, exercise physiology, and device- testing capacity. Chronic pressure-overload porcine models can reproduce concentric remodeling, fibrosis, elevated filling pressures, and preserved ejection fraction (26). Tunable soft-robotic sleeves have been used to modify left ventricular compliance and afterload in swine, creating controllable HFpEF-like hemodynamic states for device testing (27). More recently, a metabolic-syndrome Ossabaw miniature-swine model reproduced obesity, hypertension, dyslipidemia, insulin resistance, liver injury, pulmonary hypertension, elevated filling pressures, and impaired exercise capacity, providing a closer representation of cardiometabolic HFpEF (28). Even so, systematic evaluation against human diagnostic scores has shown that many large-animal models capture only subsets of the clinical syndrome (29). Cross-species translation should therefore be bidirectional and focused on mechanism rather than superficial syndrome matching. Human deep phenotyping can nominate a measurable pathway signature; experimental models can test causality and therapeutic perturbation; and model-derived signatures can then be sought again in human cohorts using physiological, imaging, biomarker, or molecular readouts. Concordance across species and measurement platforms strengthens mechanistic credibility even when no model reproduces every clinical feature (Figure 1).

**Figure 1:**
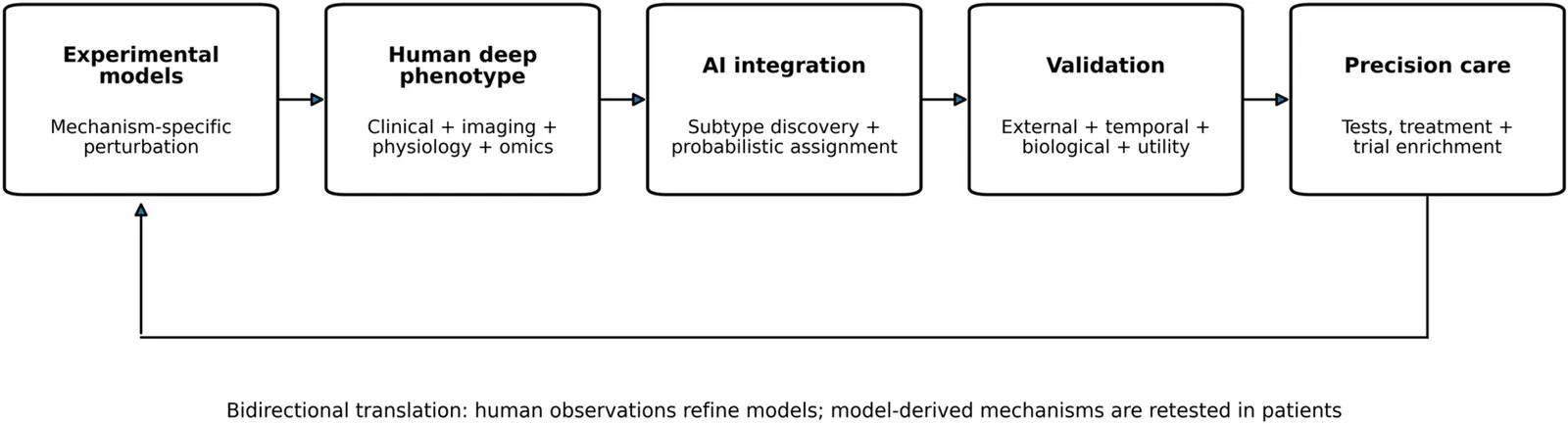
Translational framework linking experimental models to mechanistic HFpEF subtypes and precision interventions. Mechanistic domains identified in experimental systems are captured by human deep phenotyping and integrated using AI/ML to derive candidate actionable subtypes. Bidirectional translation allows human observations to refine disease models and model-derived mechanisms to generate testable therapeutic targets.

## 5 Artificial intelligence for subtype discovery and assignment

AI is particularly attractive in HFpEF because relevant information is high dimensional, nonlinear, incomplete, and distributed across modalities. Unsupervised phenomapping seeks groups without prescribing an outcome, whereas supervised models predict a defined label or event. Representation learning methods compress imaging, waveforms, text, or multi-omics into latent features; multimodal models then combine those representations. The methodological choice should follow the clinical question. Clustering is appropriate for discovering patterns, but a clinician ultimately needs an assignment rule for a new patient and an estimate of uncertainty. The modern phenomapping literature began with data-driven clustering that identified groups with distinct clinical characteristics and outcomes (30). Subsequent studies reproduced broad themes such as younger obesity-diabetes phenotypes, older vascular-renal phenotypes, and right-sided or pulmonary vascular phenotypes, although the number and composition of clusters varied with cohort selection and input variables (31–33). Recent reviews confirm that reproducibility, standardization, and clinical translation remain the principal limitations (34, 35). The instability is not necessarily algorithmic failure; it may reflect continuous biology, overlapping mechanisms, and different disease stages. Importantly, clinically based clustering has now been applied across the HFpEF continuum. Fazzini et al. demonstrated that phenomapping in preclinical heart failure with preserved or mildly reduced ejection fraction identifies clusters with distinct long-term outcomes, supporting the prognostic relevance of early phenotypic stratification (36). Uijl et al. similarly identified reproducible chronic HFpEF clusters with different clinical characteristics and prognosis (37). Extending this approach to the acute-care setting, Spagnolin et al. used machine-learning cluster analysis in hospitalized patients with HFpEF and identified low-, intermediate-, and high-risk outcome trajectories (38). Together, these studies show that phenotype clustering can retain prognostic meaning across preclinical, chronic, and hospitalized HFpEF stages; however, these groups should still be regarded as prognostic phenogroups rather than proven mechanistic endotypes. AI applied directly to echocardiographic video can detect patterns that are not fully captured by manually measured variables. A model using an apical four-chamber cine loop classified HFpEF and reclassified a substantial proportion of patients with indeterminate conventional scores (39). Follow-up analysis linked the AI classification to cardiac mortality and heart-failure hospitalization (40). These findings demonstrate potential diagnostic and prognostic value, but the latent image features require physiological explanation, and performance must be tested across vendors, acquisition protocols, demographic groups, and referral settings. For deep phenotyping, the key advance will be multimodal rather than single-modality AI. Clinical variables can provide context, imaging can define organ structure and mechanics, exercise physiology can expose reserve, and omics can anchor pathways. Early fusion concatenates all features but is vulnerable to scale differences and missing data. Late fusion combines modality-specific predictions and may be more robust when not every patient has every test. Intermediate or joint representation learning may capture crossmodal interactions, but it increases the risk that site-specific artifacts dominate the learned space. Four validation gates should be required before a data-derived subtype is called actionable. First, external validation asks whether the subtype can be assigned in an independent population. Second, temporal validation determines whether it is stable, transitions predictably, or merely reflects acute congestion. Third, biological validation tests association with a coherent physiological or molecular mechanism. Fourth, clinical-utility validation asks whether subtype knowledge improves a decision or outcome beyond standard variables. Reporting should follow contemporary prediction-model standards such as TRIPOD + AI, including transparent handling of missing data, fairness, calibration, uncertainty, and human involvement (41). Treatment- response heterogeneity is the most demanding test. A 2025 machine-learning analysis reported phenogroups with differing treatment responses, illustrating the potential of data-driven enrichment (42). Nevertheless, *post hoc* subgroup-treatment interactions can be unstable and should generate, not settle, therapeutic hypotheses. The preferred pathway is prespecified assignment of patients to a validated subtype, followed by prospective testing of a mechanism-matched intervention. Figure 2 summarizes an AI pipeline designed around this requirement.

**Figure 2:**
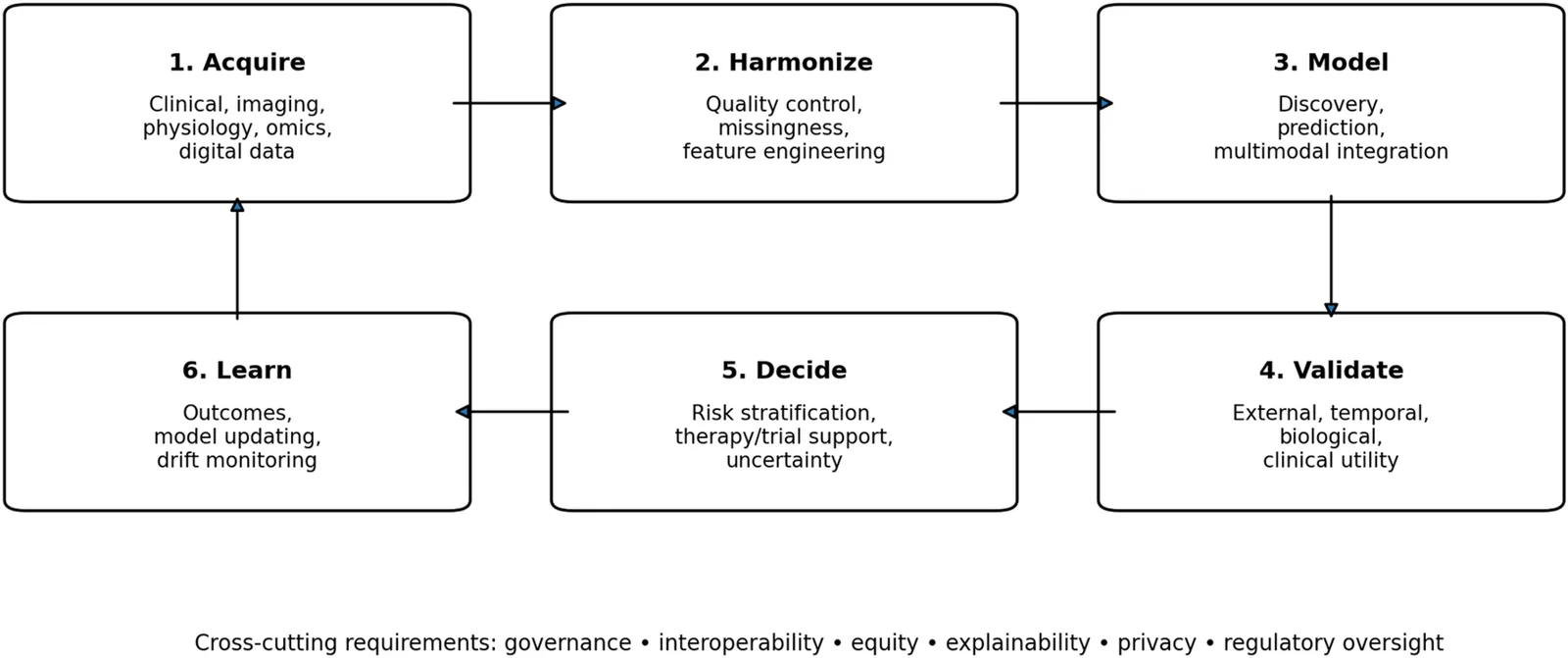
AI-enabled deep phenotyping pipeline for longitudinal precision HFpEF care. Multimodal data are harmonized and integrated for subtype discovery and outcome prediction, followed by biological and clinical validation, decision support, and continuous learning. Governance, interoperability, equity, explainability, privacy, and regulatory/ethical oversight operate across all stages.

## 6 Clinically interpretable HFpEF subtype families

A practical taxonomy should recognize that individual patients can belong to more than one subtype and that subtype probabilities can change over time. The seven families proposed here were not generated *de novo* by a clustering algorithm and are not presented as a competing consensus taxonomy. Rather, they are an author-proposed synthesis of recurrent patterns reported across foundational phenomapping, TOPCAT-derived phenogroups, subsequent cluster analyses, phenotype-profiling reviews, and contemporary mechanistic literature (9, 30–33, 43). The categories were selected because they recur across datasets, can be linked to measurable physiology or organ- system biology, and can plausibly alter the next diagnostic or therapeutic decision. They therefore function as overlapping mechanism bundles rather than mutually exclusive diagnoses. Where evidence is insufficient to demonstrate a treatment-subtype interaction, the category should be used to guide further measurement or trial enrichment rather than to prescribe therapy. Table 2 summarizes their principal features.

**Table 2:** Clinically interpretable, mechanism-informed HFpEF subtype families.

Subtype family	Dominant mechanisms	Deep-phenotype signature	Experimental anchor	Evidence/current role	Evidence gap
Cardiometabolic-inflammatory	Adiposity; insulin resistance; inflammation; volume expansion; epicardial fat	Central adiposity; diabetes; low NP; congestion; reduced function	Multi-hit metabolic rodents; Ossabaw swine	SGLT2i; weight management; incretin therapy when appropriate	Assignment beyond BMI; sarcopenia; long-term stratification
Hypertensive-vascular aging	Arterial stiffness; concentric remodeling; microvascular dysfunction	Long hypertension history; pulse pressure; LV mass/strain; exercise BP	Pressure-overload/aging; porcine afterload	Optimize BP/vascular risk; assess ischemia; avoid indiscriminate preload reduction	No validated predictive enrichment
Atrial myopathy	Atrial fibrosis/mechanical failure; AF burden; pulmonary venous hypertension	AF burden; LA strain/function; rhythm-linked symptoms	Atrial remodeling/tissue signatures	Rate/rhythm strategy; anticoagulation; obesity/sleep-apnea care	Predictors of rhythm-control benefit
Pulmonary vascular-RV	Post-capillary pressure; vascular remodeling; RV-PA uncoupling	RV function/strain; pressure-flow; PVR; exercise PH	Hemodynamic porcine models/platforms	Define hemodynamics; manage congestion/lung disease; targeted trials	Need precise enrichment for vasodilators
Cardiorenal-congestive	Venous congestion; renal dysfunction; neurohormonal activation; diuretic resistance	CKD/albuminuria; diuretic need; potassium vulnerability; recurrent congestion	CKD-metabolic/volume-loading models	Decongestion; SGLT2i; finerenone when appropriate; renal safety	Dynamic assignment and monitoring-response pathways
Peripheral-frailty	Chronotropic incompetence; skeletal-muscle dysfunction; impaired O2 extraction; sarcopenia	Low peak VO2/6MWT; weakness; frailty; peripheral extraction	Exercise/metabolic models; muscle assays	Exercise rehabilitation; nutrition; weight/anemia management	Mechanism-specific rehabilitation; durable outcomes
Specific etiologies/phenocopies	Amyloid; HCM; constriction; valve disease; high-output/storage disorders	Etiology-specific imaging; ECG; biomarkers; genetics; red flags	Disease-specific models	Dedicated etiologic diagnosis/treatment pathway	Avoid misclassification and delayed diagnosis

AF, atrial fibrillation; BMI, body mass index; BP, blood pressure; CKD, chronic kidney disease; HFpEF, heart failure with preserved ejection fraction; LA, left atrial; LV, left ventricular; PA, pulmonary arterial; PH, pulmonary hypertension; PVR, pulmonary vascular resistance; RV, right ventricular; SGLT2, sodium-glucose cotransporter 2; VO2, oxygen uptake.

### 6.1 Cardiometabolic-inflammatory subtype

Obesity-related HFpEF is characterized by more than excess body mass. Patients often have expanded plasma volume, ventricular interaction, increased epicardial adipose tissue, systemic inflammation, insulin resistance, impaired skeletal-muscle oxygen utilization, and disproportionate symptoms despite lower natriuretic peptide concentrations (44). Contemporary molecular reviews describe cardiometabolic HFpEF as an inter-organ syndrome involving adipose tissue, liver, kidney, vasculature, skeletal muscle, and myocardium (45). Deep phenotyping should therefore include body composition or waist measures, metabolic status, sleep-disordered breathing, epicardial or visceral adiposity when available, inflammatory markers, functional capacity, and congestion burden. This is currently the most therapeutically mature example of phenotype-directed care. Weight-loss therapies can improve symptoms and function, and tirzepatide has reduced worsening heart-failure events or cardiovascular death in patients with obesity-related HFpEF. However, not every patient with obesity has the same mechanism. Marked sarcopenia, advanced right-sided failure, or severe chronic kidney disease may modify the risk-benefit balance and should be captured rather than hidden within body mass index. The boundary with the cardiorenal-congestive phenotype is intentionally porous. The American Heart Association cardiovascular-kidney-metabolic (CKM) framework emphasizes that adiposity, metabolic dysfunction, kidney disease, and cardiovascular disease form an interconnected syndrome rather than independent compartments (46). In this Review, the cardiometabolic-inflammatory label denotes metabolic and adipose-inflammatory biology as the dominant signal, whereas the cardiorenal-congestive label denotes renal vulnerability, venous congestion, diuretic resistance, and recurrent volume-related events as the dominant clinical problem; patients may satisfy both profiles simultaneously. The FLOW trial further supports this overlap, because semaglutide reduced kidney and cardiovascular events in people with type 2 diabetes and chronic kidney disease, illustrating shared metabolic-renal-cardiovascular mechanisms (47). GLP-1 receptor agonists may also exert anti- inflammatory effects relevant to obesity-related HFpEF, although this remains mechanistically supportive rather than proof of a specific subtype-treatment interaction (48).

### 6.2 Hypertensive-vascular aging subtype

Long-standing hypertension, arterial stiffness, concentric remodeling, impaired ventricular-arterial coupling, chronotropic incompetence, and microvascular dysfunction commonly cluster in older patients. The dominant abnormality may be limited cardiovascular reserve rather than severe resting congestion. Deep phenotyping should include central or ambulatory blood pressure, pulse pressure, ventricular mass and geometry, strain, arterial stiffness when available, exercise blood pressure, and chronotropic response. This phenotype overlaps with cardiometabolic disease but may occur in leaner patients with vascular aging and relatively less systemic metabolic inflammation. Proximal aortic stiffening deserves particular attention because it increases pulsatile load, worsens ventricular-arterial coupling, and can limit cardiovascular reserve during exercise. Measures such as pulse-wave velocity, central pulse pressure, and their relation to myocardial deformation can therefore provide a more direct vascular phenotype than brachial blood pressure alone; contemporary work supports arterial stiffness as an intermediate phenotype linking vascular aging, comorbidity burden, diastolic dysfunction, and reduced exercise capacity (49). The therapeutic implication is rigorous control of blood pressure and comorbid vascular disease without excessive reduction of preload or perfusion. The subtype is biologically plausible but not yet supported by a dedicated successful mechanism-enriched trial. Its main value is to identify modifiable loading conditions, detect occult ischemia or microvascular dysfunction, and avoid assuming that all exertional limitation is caused by volume overload.

### 6.3 Atrial myopathy and atrial fibrillation subtype

Atrial fibrillation can be a cause, consequence, and marker of advanced HFpEF. Left atrial enlargement alone is an incomplete phenotype; atrial strain, reservoir and conduit function, rhythm burden, fibrosis, natriuretic peptide context, and exercise-related pressure responses are more informative. Left atrial myopathy may contribute to pulmonary venous hypertension even when ventricular diastolic indices are only modestly abnormal (50). Deep phenotyping can distinguish an arrhythmia-dominant patient with potentially reversible hemodynamic compromise from a patient in whom atrial fibrillation is a late marker of diffuse myocardial and vascular disease. Precision care may include aggressive rhythm assessment, anticoagulation according to established indications, treatment of sleep apnea and obesity, and consideration of rhythm-control strategies in selected patients. Atrial phenotype should also be included in trial stratification because atrial fibrillation changes heart rate, filling, natriuretic peptides, exercise reserve, and the interpretation of several diagnostic variables. Contemporary AF guidance supports an integrated AF-CARE approach with management of comorbidities and risk factors, stroke prevention, rate or rhythm control according to symptoms and clinical context, and repeated reassessment (51). In parallel, recent consensus documents conceptualize atrial cardiomyopathy as structural, electrical, and functional atrial disease that may precede or be accelerated by AF, while an HFA consensus has further framed progression from atrial dysfunction to atrial failure in heart failure (52, 53). These concepts support targeted rhythm assessment and treatment but do not yet establish a unique HFpEF atrial phenotype that predicts differential benefit from a specific rhythm-control strategy.

### 6.4 Pulmonary vascular-right ventricular subtype

Pulmonary hypertension and right ventricular dysfunction identify a high-risk HFpEF phenotype, but elevated pulmonary artery pressure does not specify mechanism. Patients may have passive post- capillary transmission, pulmonary vascular remodeling, abnormal pulmonary artery pressure-flow response during exercise, impaired right ventricular contractile reserve, tricuspid regurgitation, lung disease, or combinations of these factors (54, 55). Deep phenotyping should include right ventricular size and function, tricuspid annular and strain measures, pulmonary pressure estimates, lung function, ventilation, and—when the result will alter management invasive hemodynamics at rest or during exercise. Exercise pulmonary hypertension can reveal latent pulmonary vascular disease before severe resting abnormalities develop. Biventricular cardiac power reserve and right ventricular-pulmonary arterial coupling may better reflect the integrative limitation than resting pressure alone (55). This subtype is attractive for targeted trials, but pulmonary vasodilators cannot be assumed to benefit all patients with HFpEF and pulmonary hypertension. Enrichment should distinguish combined post- and pre-capillary physiology from isolated elevation of left-sided filling pressure.

### 6.5 Cardiorenal-congestive subtype

In cardiorenal HFpEF, chronic kidney disease, venous congestion, neurohormonal activation, diuretic resistance, anemia, and electrolyte vulnerability interact. The phenotype is dynamic: a patient may be stable with compensated renal dysfunction and later transition to recurrent congestion with escalating diuretic requirements. Deep phenotyping should include estimated glomerular filtration rate, albuminuria, potassium, iron status, serial weight and diuretic dose, venous congestion measures, and the relation between renal function and decongestion. The clinical goal is not to protect a laboratory creatinine value at the expense of persistent congestion. Rather, subtype-informed management should balance adequate decongestion, renal perfusion, potassium safety, and disease-modifying therapy. Remote hemodynamic or digital monitoring may be most useful in patients with repeated, predictable congestive trajectories, but the benefit of intensive monitoring depends on a responsive treatment pathway.

### 6.6 Peripheral limitation, skeletal-muscle dysfunction, and frailty subtype

Some patients have severe exercise intolerance out of proportion to resting cardiac abnormalities. Reduced peak oxygen uptake may reflect chronotropic incompetence, impaired peripheral oxygen extraction, skeletalmuscle mitochondrial dysfunction, obesity, anemia, pulmonary disease, or frailty. Invasive or non-invasive exercise phenotyping can separate inadequate cardiac output reserve from peripheral limitation; exercise-related lung congestion further refines the mechanism (56). Functional testing should include gait speed or frailty measures, strength, peak oxygen uptake, ventilatory efficiency, and—when feasible—body composition. Exercise training and caloric restriction have improved exercise capacity and quality of life in obese older patients with HFpEF (57), and a contemporary meta-analysis supports exercise training as an effective component of care (58). The treatment signal is therefore actionable even when the molecular mechanism is not fully defined. A frailty label should not be used to exclude patients from therapy; it should trigger adapted rehabilitation, nutritional assessment, fall-risk management, and realistic patient-centered outcomes.

### 6.7 Specific etiologies and phenocopies

A precision framework must protect against misclassifying specific diseases as nonspecific HFpEF. Cardiac amyloidosis, hypertrophic cardiomyopathy, constrictive pericarditis, significant valvular disease, high-output states, and infiltrative or storage disorders require distinct diagnostic and therapeutic pathways. Red flags from electrocardiography, imaging, family history, extracardiac manifestations, and biomarker patterns should override generic phenogroup assignment. AI may help identify subtle patterns, but it should increase rather than reduce etiologic vigilance.

## 7 From phenogroups to precision care

Precision care in HFpEF should be layered rather than exclusive. The first layer is evidence-based therapy that applies broadly to eligible patients. Sodium-glucose cotransporter 2 inhibitors reduce heart- failure events across the preserved-ejection-fraction spectrum and should not be withheld while waiting for sophisticated phenotyping (59, 60). Finerenone reduced total worsening heart-failure events and cardiovascular death in patients with mildly reduced or preserved ejection fraction, providing an additional option particularly relevant to patients with mineralocorticoid- receptor activation, diabetes, kidney disease, or inflammatory-fibrotic features, while requiring potassium and renal monitoring (61). For clarity, therapeutic evidence in this Review is separated into three levels. Level A comprises treatments with demonstrated benefit across broadly enrolled HFpEF populations and should be offered on the basis of guideline eligibility rather than phenotype assignment. Level B comprises interventions tested prospectively in phenotype-enriched populations, such as incretinbased therapy in obesity-related HFpEF. Level C comprises biologically plausible or clinically reasonable phenotype-guided strategies for which no validated treatment-subtype interaction has yet been demonstrated. This hierarchy is also reflected in Table 2 and is intended to prevent routine comorbidity management from being misinterpreted as evidence of predictive endotyping. The second layer is phenotype-directed treatment. In obesity-related HFpEF, semaglutide improved symptoms, physical limitations, exercise function, and weight in patients with and without diabetes (62, 63). Tirzepatide subsequently reduced worsening heart-failure events or cardiovascular death and improved health status in patients with HFpEF and obesity (64). These trials are important because they targeted a clinically recognizable phenotype with a mechanism- directed intervention and patient- centered outcomes. They do not establish that incretin-based therapy is appropriate for every HFpEF patient, nor do they remove the need to assess sarcopenia, gastrointestinal tolerance, cost, and long- term adherence. The third layer is hypothesis-driven targeting of less mature subtypes. Mineralocorticoid receptor antagonism with spironolactone produced a neutral primary result in TOPCAT overall, although regional and subgroup analyses have continued to inform trial design (65). Sacubitril-valsartan narrowly missed its primary endpoint in PARAGON-HF but suggested heterogeneity according to ejection fraction and sex (66). These examples show why retrospective subgroup findings require prospective confirmation. A phenogroup may identify prognosis without predicting treatment response, and a statistically significant interaction may not reproduce. Symptom and functional outcomes should be treated as core endpoints, not secondary decoration. This position is supported by patient-preference data showing that many people with heart failure place substantial value on quality of life relative to longevity, and by the 2025 HFA/EAPC scientific statement emphasizing systematic assessment of quality of life in both trials and clinical care (67, 68). Dapagliflozin improved symptoms and physical limitations in PRESERVED-HF (69), while phenotype-enriched obesity trials such as STEP-HFpEF and SUMMIT incorporated Kansas City Cardiomyopathy Questionnaire measures as major efficacy outcomes. These precedents demonstrate that meaningful treatment benefit can be captured through health status and function even when resting ejection fraction changes little. Precision trials should therefore match endpoints to the proposed mechanism: congestion events for a volume-sensitive subtype, peak oxygen uptake for a reserve-limited subtype, rhythm burden for an atrial subtype, or health status and physical function for an obesity-frailty phenotype. A practical clinic can implement a minimum viable deep phenotype without universal multi-omics. It should include: standardized clinical and trajectory data; high-quality echocardiography with left atrial, right ventricular, and strain assessment when available; natriuretic peptide interpreted in the context of obesity and atrial fibrillation; renal, metabolic, hematologic, and inflammatory markers; functional assessment; and targeted exercise, invasive, CMR, genetic, or molecular testing when the result is likely to change a decision. The output should be a probabilistic mechanism profile, not a single permanent label.

## 8 Phenotype-enriched trials and implementation roadmap

The principal advantage of deep phenotyping may be improved trial design. Prognostic enrichment selects patients likely to experience events, reducing sample size, but does not ensure biological responsiveness. Predictive enrichment selects patients whose mechanism is targeted by the intervention. The two should not be confused. A trial of a pulmonary vascular therapy, for example, should require evidence of abnormal pulmonary vascular resistance or pressure-flow response, not simply an elevated echocardiographic pulmonary pressure estimate. A phenotype-enriched trial should prespecify the assignment algorithm, allowable overlap between subtypes, the timing of phenotyping relative to decongestion, and how missing modalities are handled. It should also include a standard- care control because a mechanism-matched intervention is added to, not substituted for, established therapy. Adaptive platform designs could test multiple interventions across subtype strata and update assignment rules as evidence accumulates. However, adaptation must be statistically governed and protected against algorithm drift. Implementation requires a common data model and a tiered testing pathway. The following is explicitly proposed by the author as a practical, testable clinical algorithm rather than a validated guideline. Step 1 (Tier 1) is performed in all patients with suspected or established HFpEF and includes clinical trajectory, comorbidities, ECG/rhythm burden, natriuretic peptides interpreted in context, renal-metabolichematologic laboratory testing, and comprehensive echocardiography including left atrial and right ventricular assessment. Step 2 asks whether the dominant mechanism is sufficiently clear to alter management. If yes, evidence-based therapy is initiated and the phenotype is reassessed longitudinally. If no, or if symptoms are disproportionate to resting findings, Step 3 (Tier 2) adds targeted exercise echocardiography or CPET, CMR, vascular stiffness or pulmonary assessment, and structured digital follow-up according to the unresolved mechanism. Step 4 (Tier 3) is reserved for cases in which a highcost or invasive measurement is likely to change diagnosis, trial eligibility, or a mechanism-targeted intervention; it may include invasive exercise hemodynamics, proteomics, metabolomics, genomics, or tissue/single-cell studies in specialized cohorts. At every step, specific etiologies and phenocopies must be actively excluded, and the final output should be a probabilistic mechanism profile with explicit uncertainty rather than a permanent categorical label. The same core variables should be collected across tiers to permit transportability. Bias and access are central concerns. HFpEF disproportionately affects older adults, women, people with obesity, and patients with multimorbidity, groups often underrepresented or technically challenging in imaging and exercise studies. An AI model can amplify referral bias, differences in test availability, and missingness associated with socioeconomic status. Fairness assessment should therefore examine calibration and clinical consequences across sex, age, body size, race and ethnicity where legally and ethically appropriate, renal function, rhythm, and care setting. A model should be allowed to abstain when uncertainty is high. The proposed development sequence is: define the clinical decision; specify the minimum phenotype needed; discover candidate subtypes in a deeply characterized cohort; create a portable assignment model; validate externally and longitudinally; connect the subtype to biology through physiology, omics, or cross-species evidence; and test decision utility prospectively. Reviews of precision medicine in HFpEF have reached a similar conclusion: therapeutic progress is now sufficient to make better patient selection a practical priority rather than a purely descriptive exercise (70). Recent evidence published in 2024-2026 substantially strengthens the deep-phenotyping framework. A large multi-omics analysis integrated clinical, phenomic, proteomic, metabolomic, and genetic data to identify molecularly grounded HFpEF signatures and demonstrated the feasibility of reduced, potentially portable feature sets (71). Exercise stress echocardiography has identified pathophysiologically distinct clusters that are not apparent from resting assessment alone (72), while contemporary reviews of invasive haemodynamics emphasize its role in resolving intermediate diagnostic probability and defining exercise-induced phenotypes (73). Updated phenotype-profiling syntheses now connect cardiometabolic, atrial, vascular, pulmonary vascular-right ventricular, and cardiorenal patterns with therapeutic implications (43). Longitudinal analyses further show that phenotype assignment can change between acute decompensation and the stable phase, reinforcing the need for repeated rather than one- time classification (74). Additional recent studies reinforce the value of molecular and AI-enabled phenotyping. In VITALITY- HFpEF, plasma proteomics identified biologically distinct profiles associated with clinical phenotype and functional outcomes, supporting the use of circulating protein signatures as intermediate phenotypes (75). The PARAGON-HF proteomic substudy similarly demonstrated the potential of large-scale aptamer proteomics for biomarker discovery and pathway-level characterization in HFpEF (76), while urinary proteomic profiling has linked non-invasive molecular signatures with prognosis (77). Metabolomic studies have also moved beyond descriptive profiling: targeted analysis in PRESERVED- HF identified treatment-related metabolic changes with dapagliflozin (78), and a large 2025 plasma metabolomics study demonstrated metabolic signatures that distinguish preserved from reduced ejection-fraction heart failure (79). Translationally, integrated systems-biology work published in 2025 connected mitochondrial dysfunction, substrate metabolism, and transcriptomic remodeling in an experimental HFpEF model (80), while a 2026 olink study identified candidate cardiometabolic proteins with diagnostic potential in human HFpEF (81).

These converging molecular datasets provide increasingly rich inputs for multimodal subtype discovery, but clinical adoption will require external validation, assay standardization, and demonstration that biomarker-defined groups improve therapeutic decisions. Contemporary reviews by Epelde have emphasized both the broader transition toward precision medicine in heart failure (82) and the potential role of artificial intelligence in diagnostic integration, risk prediction, treatment personalization, and longitudinal monitoring (83). Together with HFpEF-specific phenotyping evidence, these observations support a framework in which AI is used to integrate rather than replace mechanistic clinical reasoning.

## 9 Discussion

Deep phenotyping offers a route out of the false uniformity created by a preserved ejection fraction, but it also carries a risk of replacing one oversimplification with an opaque algorithm. The field has already shown that data-driven clusters can identify groups with different characteristics and outcomes. What remains uncommon is evidence that a cluster is reproducible, biologically coherent, assignable to an individual patient, and predictive of a better therapeutic choice. Those criteria should define the next generation of HFpEF studies. The most convincing current precision- care example is cardiometabolic HFpEF. Obesity, metabolic dysfunction, inflammation, volume expansion, epicardial adiposity, and physical limitation form a recognizable mechanism bundle, and phenotype-enriched trials of semaglutide and tirzepatide have produced clinically meaningful benefits. Even here, heterogeneity persists, and body mass index alone is an inadequate assignment tool. The phenotype should be refined using body composition, congestion, function, renal status, and frailty. Other subtypes are at different stages of maturity. Atrial myopathy, pulmonary vascular-right ventricular disease, and cardiorenal congestion have strong physiological plausibility and clear prognostic relevance, but dedicated predictive enrichment is less developed. Peripheral and frailty phenotypes have actionable rehabilitation strategies but incomplete molecular definition. Hypertensive- vascular aging is common and clinically intuitive, yet its boundaries and targeted treatment beyond excellent risk-factor management remain uncertain. These differences should be made explicit rather than presenting all subtypes as equally validated. Experimental models can accelerate maturation by providing causal and tissue-level evidence. The correct goal is not a universal HFpEF animal model. It is a portfolio of models, each mapped to a human mechanism signature and evaluated with comparable physiological and molecular readouts. Human single-cell atlases and deeply phenotyped registries can become reference standards against which model fidelity is quantified. Conversely, perturbation signatures from models can identify human patients most likely to share the targeted pathway. The article has limitations. It is a narrative, not systematic, review and may not include every emerging model or algorithm. The subtype families are a clinically oriented synthesis rather than a validated consensus taxonomy. Several cited treatment-response observations arise from subgroup or *post hoc* analyses and should not be interpreted as prescribing rules. Finally, rapidly evolving AI and omics methods may outpace conventional publication cycles, making transparent updating and independent validation essential. Despite these limitations, a clinically useful direction is clear. HFpEF care should combine broadly effective treatment with targeted investigation of dominant mechanisms. Deep phenotyping should be performed in tiers, escalation should be justified by decision impact, and AI should provide probabilities and uncertainty rather than an unexplained permanent label. The success metric is not a higher- dimensional description of HFpEF; it is better selection of tests, treatments, and trials.

## 10 Conclusion

HFpEF is best understood as a family of overlapping, evolving syndromes rather than a unitary disease defined by ejection fraction. Deep clinical, physiological, molecular, and digital phenotyping can identify mechanism bundles, while experimental models provide causal anchors and AI supplies tools for multimodal integration. Translation will require a higher standard than statistical separation: external reproducibility, biological coherence, temporal validity, and demonstrated clinical utility. A layered strategy—universal evidence-based therapy, phenotype-directed management, and prospective reassessment—can make precision HFpEF care practical before every patient undergoes multi-omics. The immediate research priority is to test validated, portable subtype assignments in mechanism-enriched clinical trials and to determine whether they improve outcomes compared with contemporary guideline-based care.
